# Long-term high-dose immunoglobulin successfully treats Long COVID patients with pulmonary, neurologic, and cardiologic symptoms

**DOI:** 10.3389/fimmu.2022.1033651

**Published:** 2023-02-02

**Authors:** John S. Thompson, Alice C. Thornton, Timothy Ainger, Beth A. Garvy

**Affiliations:** ^1^ Division of Pulmonary and Critical Care Medicine, Department of Internal Medicine, College of Medicine, University of Kentucky, Lexington, KY, United States; ^2^ Division of Infectious Diseases, Department of Internal Medicine, College of Medicine, University of Kentucky, Lexington, KY, United States; ^3^ Department of Neurology, University of Kentucky, College of Medicine, Lexington, KY, United States; ^4^ Department of Microbiology, Immunology and Molecular Genetics, College of Medicine, University of Kentucky, Lexington, KY, United States

**Keywords:** immune perturbation, autoimmune, fatigue, brain fog, microvasculitis, neuropathy

## Abstract

**Introduction:**

Long COVID is the overarching name for a wide variety of disorders that may follow the diagnosis of acute SARS-COVID-19 infection and persist for weeks to many months. Nearly every organ system may be affected.

**Methods:**

We report nine patients suffering with Long COVID for 101 to 547 days. All exhibited significant perturbations of their immune systems, but only one was known to be immunodeficient prior to the studies directed at evaluating them for possible treatment. Neurological and cardiac symptoms were most common. Based on this data and other evidence suggesting autoimmune reactivity, we planned to treat them for 3 months with long-term high-dose immunoglobulin therapy. If there was evidence of benefit at 3 months, the regimen was continued.

**Results:**

The patients’ ages ranged from 34 to 79 years—with five male and four female patients, respectively. All nine patients exhibited significant immune perturbations prior to treatment. One patient declined this treatment, and insurance support was not approved for two others. The other six have been treated, and all have had a significant to remarkable clinical benefit.

**Conclusion:**

Long-term high-dose immunoglobulin therapy is an effective therapeutic option for treating patients with Long COVID.

## Introduction

SARS-COVID-19 has now affected the world for more than 2 years. Although the majority of patients eventually recover after the acute illness, it is now estimated that greater than 20% continue to have lingering symptoms that may affect their quality of life (1–4). Some reports suggest that, when these persist for greater than 4 weeks post-acute infection, the term Long COVID may be given. However, the initial symptoms are sometimes fading by that time. On the other hand, if the symptoms are continuing for greater than 12 weeks, the chances are greater that they will persist and constitute the Long COVID syndrome. Dyspnea and fatigue are the most common symptoms, but post-exertional fatigue and cognitive dysfunction are also common. Moreover, major neurological symptoms (5), cardiovascular diseases (6), anxiety, depression, stroke (7), and peripheral neuropathies (8, 9) are among the many Long COVID disorders affecting nearly every organ. As with the major symptoms occurring during the acute phase (9, 10), there is increasing evidence that many of these Long COVID disorders are related to the immune system (9–12), including symptoms of peripheral neuropathy (5) and autoimmune encephalitis (13–16). Many reports have documented the wide variety of mild to severe symptoms that arise and persist long after an acute infection with SARS-COVID-19. However, there are fewer reports on the various treatments and their effects on the severity and duration of these symptoms. No consensus for the underlying pathologic mechanisms or treatment of Long COVID has emerged as yet.

Based on the evidence that immune dysregulation may be a major factor in some COVID-related disorders, high-dose intravenous immunoglobulin (IVIG) has been administered alone or in conjunction with immunosuppressive drugs as part of the treatment during the acute phase (17–22).

Recently, ten patients with Long COVID have been referred to us—all of whom have exhibited increased to decreased immune dysregulation. Although the immunologic perturbations differed within the group, only one of them was known to be immunodeficient prior to the studies directed at evaluating them for possible treatment of Long COVID. Based on this data and other evidence suggesting an autoimmune perturbation, we have treated six of these patients with high-dose immunoglobulin so far, and all have had a significant to remarkable benefit (see the following brief case reports).

In this study, we present the results of the administration of high-dose immunoglobulin initiated 101 to 547 days after their first COVID-19 infection. Of the other four untreated patients, one patient declined this treatment, and we have been unable to get insurance approval for this regimen in three patients.

## Materials and methods

### Patients

This report presents the evaluation and treatment results of nine patients referred to our adult immunology clinic between December 4, 2020 and March 16, 2022 with a diagnosis of Long COVID. In addition to their history and physical examination, the following tests were routinely performed: antibody response to rubeola, hepatitis A, mumps, and pneumococcal serotypes if they had been vaccinated; antibody to COVID nucleocapsid; antibody to COVID spike protein if they had been vaccinated with an RNA vaccine; levels of total IgA, IgM, IgG, and IgG subclasses IgG1, IgG2, IgG3, and IgG4; evaluation of lymphocyte subsets by flow cytometry; erythrocyte sedimentation rate, and C-reactive protein. If the history, clinical examination, and prior laboratory tests suggested, anti-nuclear antibody, rheumatoid factor, antineutrophil cytoplasmic antibody panel, and high-sensitivity troponin were commonly tested as well. Depending on the clinical course, all of the above-mentioned tests—except the antibody responses—were repeated periodically during the administration of the high-dose immunoglobulin.

### Treatment plan

Unless stated otherwise, the planned treatment was to administer 0.5 g/kg IVIG every 2 weeks for a 3-month trial and, if clinical benefit was observed at that time, to continue the regimen. In two patients, the dose and the frequency had to be modified to obtain insurance approval (patients 2 and 5).

Extensive neuropsychiatric testing was performed on some patients. There was no attempt to alter the medications that the patients were on before initiating the IVIG. Although they may have received courses of high-dose steroids in the past, none had been treated for at least 2 months and none were on steroids at the time of IVIG. One patient with autoimmune encephalitis was receiving 1.0 gm mycophenolate daily, but it had not appreciably altered his major neuropsychiatric symptoms. Another patient with Wegener’s granulomatosis had received methotrexate and rituximab prior to the diagnosis of COVID-19 but neither within 6 months of the treatment with IVIG.

## Results

### Summary of the six patients who have received high-dose immunoglobulin therapy

#### Patient 1

A 62-year-old professional male patient developed dyspnea associated with viral pneumonia, tested PCR-positive for SARS-COVID-19, and admitted to the University of Kentucky hospital on March 26, 2020. Details of this and later admissions have been published in detail (13) and are briefly reviewed herein. He did not require ventilation, improved on therapy, and was discharged to home after testing PCR-negative. After a self-quarantine for 14 days and again testing negative, he resumed with his normal social activities. However, on June 4, 2020, he was readmitted with confusion, difficulty with word finding, trouble initiating tasks, waxing and waning lucidity, and loss of memory. He was discharged 2 days later but continued to decline and was admitted again on June 10, 2020 because of increasing difficulty in performing daily tasks, making calculations, and keeping his balance. Importantly, “out-of-character episodes” that were sometimes violent, increased paranoia, anxiety, and further loss of awareness and memory were observed. The results of CT of the head and neck demonstrated stable 3.5- and 2.0-cm pseudoaneurysms. The MRI examination revealed no acute pathology, but a small infarct was present in the right medial thalamus. An EEG video was reported as suggestive of severe diffuse encephalopathy, and a PET/CT taken on June 15, 2020 demonstrated abnormal mixed brain hypo- and hypermetabolism in many areas of the brain. The results of a repeat PET/CT on June 24, 2020 noted further decreases in brain metabolism. He received IVIG daily from June 14–25, 2020, and initially there was a definite improvement in his mental status. He was discharged on June 25, 2020, and for a while the improvement continued. Unfortunately, he progressively redeveloped severe neuropsychiatric symptoms including psychoses, hallucinations, disinhibition/impulsivity, and frequent severe panic attacks. A repeat neuropsychiatric testing on September 04, 2020 continued to show areas of severe disability in several brain functions. He was readmitted on November 16, 2020, and in the course of this and previous admissions, a very complete autoimmune evaluation for the encephalitis was performed. All tests were normal or negative, with the exception of ANA at 1:160. He received a second course of IVIG and started on 1,000 mg mycophenolate a day and was discharged on November 20, 2020.

He was referred to Immunology on December 4, 2020 (**Table 1**). During the review of history and examination, the most striking feature was his almost complete failure to participate in the process. He responded correctly to commands, but his wife provided most of the information. The results of the immunologic tests demonstrated that both the total IgG and IgG1 subclasses were elevated, and qualitative anti-COVID IgG was reported as very high (ANA, 1:160). High total IgG and IgG subclass 1 are features of several classic autoimmune disorders. These laboratory results, the clinical diagnosis of autoimmune encephalitis, and the partial response to IVIG suggested that a 3-month trial of high-dose IVIG could be a way to mitigate this patient’s severe neuro/psychiatric disorder. On January 16, 2021 (296 days after the acute COVID-19 infection, **Table 1**), he was begun on 0.5 g/kg IVIG every 2 weeks. Mycophenolate was continued at 1,000 mg/day. He has tolerated both treatments with no significant adverse effects. Within 2 months, the paranoia, hallucinations, disinhibition/impulsivity, and frequent severe panic attacks had largely stopped, but there were still periods of waxing and waning participation in his surroundings and social events. The trial has been continued up to the present. Currently, he conducts most of the interim report without the help of his wife, and she confirms that he is almost back to normal. However, the results of neuropsychiatric testing on March 1, 2022 continued to show significant defects in verbal learning and reading speed. The most recent laboratory evaluations demonstrated a decrease to normal in the total IgG and IgG subclass 1 and persistently low IgM immunoglobulins (**Table 2**). The administration of mycophenolate has been stopped, a slow taper of the immunoglobulin dosage is now underway, and there has been no relapse of his symptoms.

**Table 1:** Demographics and therapy.

Patient	Age	Sex	Pre-existing conditions	Acute Covid onset date	Symptoms	Immune perturbations	Ig q/2 wks.	Onset to Ig (days)!
# 1	63	male		03/25/2020	encephalitis fatigue digital tingling	high IgG high IgG1 low IgM	0.5g/kg	298
# 2	50	female	hypertension hyperlipidemia	03/04/2020	brain fog* chest pain fatigue dyspnea	low IgG2 low IgG3	0.5g/kg.	547
# 3	38	female	sinusitis	03/11/2020	brain fog* dyspnea nausea/vomiting tingling left side	low IgG2 low IgG3	0.4g/kg	452
# 4	73	male	Hypertension sinusitis	01/02/2021	brain fog* chest pain dyspnea fatigue	low IgM low CD8 HLA-DR on T cell 15%	0.5g/kg.	258
# 5	34	female	asthma	01/02/2022	brain fog* tingling hands numbness feet	low IgG2 low IgG3	0.5g/kg**	101
# 6	39	male	Wegener’s sinusitis	11/08/2021	dyspnea severe hypoxia pneumonia x3 fatigue	low IgG, M and A very low CD4, CD8 and CD19. HLA-DR on T cells CD4 14%, CD8 36%	0.5g/kg.	140

* brain fog = a term encompassing decreased memory, difficult word finding, a sense of distance/fog from surroundings.

** 3 week frequency between infusions.

! = interval between the date of acute Covid and first day of immunoglobulin infusion.

**Table 2:** Laboratory data.

Patient	Date of tests	Ig Profile (mg/kg)	IgG subclasses (mg/kg)	Lymphocyte subsets (cells/ul)	Antibody tests*	Hemogram**	Covid Ig ***
# 1	12/04/2020 (pre-Ig Rx)	IgG 1,781 IgM 46 IgA 97	IgG1 950 IgG2 569 IgG3 48 IgG4 69.8	CD3 1,053, CD4 745 CD8 298, CD19 141 CD16/56 79 HLA-DR T < 10%	rubeola + mumps + pneu. 4/14+ ANA 1:160	WBC 6.34 cells/ul Hgb 14.1 g/dl Hct 41.4% Plat. 225 K cells/ul	Covid – Spike +
	06/15/2022 (current Ig Rx)	IgG 1,168 IgM 33 IgA 76	IgG1 622 IgG2 465 IgG3 29 IgG4 49.3	CD3 1,000, CD4 663 CD8 167, CD19 104 CD16/56 79 HLA-DR T 4%		WBC 7.93 cells/ul HgB 14.6 g/dl Hct 42.8% Plat. 174 K cells/ul	
#2	12/18/2020 (pre Ig Rx)	IgG 923 IgM 211 IgA 194	IgG1 489 igG2 229 IgG3 10 IgG4 19.4	CD3 1,703, CD4 1,136 CD8 498, CD19 362 CD16/56 276 HLA-DR T <10%	rubeola + mumps eq. Hepatitis A +	HgB 12.5 g/dl Hct 39.6% Plat.312 K cells/ul	Covid—
	04/27/2922 (current Ig Rx)	IgG 1,800 IgM 220 IgA 100	IgG1 1,110 IgG2 634 IgG3 22 IgG4 49.2	CD3 1,563, CD4 1,080 CD8 465, CD19 153 CD16/56 314 HLA-DR T < 19%		WBC 5.50 cells/ul HgB 14.0 g/dl Hct 40.4% Plat.306 K cells/ul	
#3	12/18/2020 (pre Ig Rx)	IgG 820 IgM 225 IgA 397	IgG1563 IgG2 116 IgG3 13 IgG4 19.5	CD3 1,540, CD4 1,274 CD8 228, CD19 294 CD16/56 263 HLA-DR < 10%	rubeola eq. mumps + ANA!:80	WBC 5.05 cells/ul HgB 14.1 g/dl Hct 42.7% Plat. 262 K cells/ul	Covid—
	04/27/2022 (current Ig Rx)	IgG 2,016 IgM 220 IgA 199	IgG1 1,101 IgG2 626 IgG3 28 IgG4 50.4			WBC 3.03 cells/ul HgB 12.5 g/dl Hct 36.8% Plat.200 K cells/ul	
#4	09/03/2-21 (pre Ig Rx)	IgG 1,163 IgM 35 IgA 179	IgG1 476 IgG2 404 IgG3 24 IgG4 41.6	CD3 1,299, CD4 1,141 CD8 135, CD19 153 CD16/56 268 HLA-DR T 15%	Hepatitis A + pneu. 7/14 +	WBC 5.71 cell/ul HgB 14.9 g/dl Hct 45.1% Plat, 281 K cells/ul	Covid – Spike +
	06/13/2022 (current Ig Rx)	IgG 215 IgM 34 IgA 225		CD3 1,986, CD4 1.717 CD8 239, CD19 166 CD 16/56 495 HLA-DR T 17%		WBC 6.89 cells/ul HgB 14.4g/dl Hct 43.1% Plat. 284 K cell/ul	
#5	03/05/2022 (pre Ig Rx)	IgG 960 IgM 145 IgA 145	IgG1 602 IgG2 157 IgG3 21 IgG4 18.7	CD3 1,145, CD4 884 CD8 487, CD19 260 CD 16/56 HLA-DR T 9%	rubeola + mumps +	WBC 6.93 cells/ul HgB 13.9 g/dl Hct 40.6% Plat. 329 K cells/ul	Covid – Spike +
#6	01/07/2022 (pre Ig Rx)	IgG 429 IgM 7 IgA 73		CD3 21, CD4 17 CD8 9, CD19 0 CD16/56 50 HLA-DR T CD4 36%, CD8 14%		WBC 9.16 cells/ul HgB 13.9 g/dl Hct 48.9% Plat. 193 K cells/ul	Covid – Spike -
	06/17/2022 (current Ig Rx)	IgG 2,306 IgM 11 IgA 61		CD3 276, CD4 200 CD8 63, CD19 107 CD16/56 76 HLA-DR T 28%		WBC 5.56 cells/ul HgB 14.5 g/dl Hct 43.4% Plat. 210 K cells/ul	

* + = positive; **Hemogram: WBC, white blood count; HgB, hemoglobulin; Hct, hematocrit; Plat., platelet.

** IgG antibody tests: + = positive, - = negative; pneu. = pneumococal serotypes.

***+ = positive, - = negative; covid = nucleocapsid IgG; spike = immunized to one of the RNA vaccines,

Ig Rx = immunoglobulin treatment.

#### Patient 2

A 50-year-old female patient with a past medical history of controlled hypertension and hypothyroidism was diagnosed with COVID-19 in March 2020. The initial symptoms included loss of taste and smell, malaise, non-productive cough, chills, severe headaches, memory loss, confusion, and profound fatigue. She was not hospitalized, and the symptoms moderately improved in 2 to 3 weeks. However, as she began to recover, chest pain was noted not only after coughing but also after exercise, and she continued to experience shortness of breath and fatigue even with limited exercise as well as simple daily chores. Although metoprolol helped relieve the pain, fatigue, “brain fog, difficulty in word finding”, and shortness of breath persisted. Her full pulmonary function tests were normal, and she did not desaturate on a 6-min walk, but she could not complete the walk because of shortness of breath. After an extensive cardiac workup, a final diagnosis of cardiac microvasculitis was determined. Although she had been able to continue working, the fatigue continued to be characterized by having to sleep for 12 or more hours, the chest pain was recurring almost daily, and she continued to have difficulty recalling words and remembering events.

A diagnosis of Long COVID was established, and she was referred to Immunology on January 29, 2021—almost 2 years after the acute COVID infection (**Table 1**). In addition to the above-mentioned history, she denied any history of recurrent infections, skin rashes, or neurological symptoms. On physical exams, her vital signs were normal, she was alert, and she had little trouble recalling historical and other events. The immunologic testing results demonstrated low IgG2 and 3 subclass immunoglobulins, negative anti-COVID nucleocapsid IgG, and an equivocal response to rubeola (**Table 2**).

Since there was no past history of repeated childhood or adult recurrent infectious diseases, the low IgG2 and IgG3 suggested that they had been caused by the COVID infection. Taking this hypothesis and the prolonged symptoms of Long COVID into consideration, she was begun on 0.5 g/kg IVIG every 2 weeks on September 27, 2021—547 days after the documented onset of acute COVID. With the exception that she has required a port for the infusions, there have been no adverse events reported. She now reports that the chest pain and the word finding difficulty are essentially absent and the fatigue is much less. She states that, with the exception of some mild decrease in long-term memory, she is back to her pre-COVID self.

#### Patient 3

A 38-year-old nurse was being routinely tested, because she was part of a research study, when in late March 2020 she tested COVID-19-positive. She was asymptomatic but quarantined. At 2 days later, she reported that she did have a sore throat and mild fatigue but still felt generally well. At that time, there was loss of taste and smell, as well as fever, but no significant malaise or cough. At 5 days later, she reported increasing fatigue, unrelenting nausea, periodic diarrhea, shortness of breath, and O_2_ levels in the low 90s. Thus, 40 mg bid prednisone was initiated, but it did not help. On June 11, 2020, periodic tachycardia and worsening shortness of breath were noted. The PCR test had become negative. Over the forthcoming days, these symptoms increased, and she began to note word finding difficulties, night sweats, and tingling of the left side of her face. Cognition further deceased progressively, and she was put on medical leave. It is worth noting that an anti-SARS-COVID nucleocapsid IgG test was negative at this time and again at 1 month later. She was seen by cardiology, neurology, and pulmonology experts. Her brain MRI and cardiac catheterization results were normal. The complete pulmonary function tests showed only mild obstruction, which was not severe enough to account for the marked dyspnea. Depression had become an issue, and she began medication with some benefit for this symptom but not for the primary COVID-related symptoms.

She was referred to Immunology on January 13, 2021 (**Table 1**). The past medical history of recurrent ear infections and sinusitis as a child was noted as well as the fact that the sinusitis and bronchitis were still periodically recurring prior to the time of her COVID infection. All of the above-mentioned COVID-listed symptoms were still present. Her physical exam result was essentially normal. She continued to report shortness of air and, particularly, chronic fatigue and cognition difficulties. The result of a pertinent laboratory workup was normal, with the exception of low IgG2 and IgG3 immunoglobulin subclasses (**Table 2**). Her antibody to mumps was equivocal but positive to rubeola and hepatitis A.

After a delay relative to insurance coverage, she was started on 0.4 g/kg immunoglobulin every 2 weeks on November 17, 2021—which was 452 days after the initial COVID infection. At 2 months later, she reported that she had much less fatigue and less difficulty in word finding but stated that she did not feel like she was ready to return to work. She received two doses of Pfizer vaccination and developed a positive anti-spike titer.

Unfortunately, she required a port, and it became infected with *Mycobacterium fortuitum*. The port was removed, and she was admitted to the hospital with an enlarging wound and a fever that required IV antibiotics and oral levofloxacin for an additional 8 weeks. She experienced joint pains but no joint swelling. During this time, she tested positive by PCR for the second time to COVID, and there was a recurrence of severe fatigue, dyspnea, and drenching night sweats. In addition, she noted several newly occurring raised, reddish, and somewhat painful lesions in her scalp and at the hair line. The repeated tests revealed an elevated sedimentation rate and IgM immunoglobulin that had not been present at the time of the acute COVID symptoms. She reported that her mother has scalp psoriasis and her maternal grandmother had more generalized psoriasis. Her primary care physician had noted her thyroid-stimulating hormone (TSH) to be rising to an abnormally high level and started her on 25 μg Synthroid. Within 2 weeks, she reported to have an increase in energy and less fatigue and that she was definitely better, which corresponded to her TSH returning to a normal level.

#### Patient 4

A 73-year-old male patient with a past medical history of rheumatic fever as a child, hypothyroidism, hypertension, bradycardia, gout, and recurrent sinus infections developed COVID symptoms on January 2, 2021. He was admitted on January 4, 2021 because of increasing dyspnea. Hypoxia developed, and he required O_2_ but not mechanical ventilation. His condition improved, and he was discharged, but in the months since then, he suffered dyspnea on exertion, dizziness, double vision, myalgias, persistent fatigue, and chest pain. Prior to COVID, he had been physically active with long walks and weightlifting, but he now noted dyspnea on walking as little as one block and had to take 2- to 3-h naps each afternoon because of fatigue. The MRI result showed only “age-related” changes in the brain, and his complete pulmonary function tests were normal. Because of periodic left chest pain and an elevated troponin level, he had an extensive cardiac evaluation. His ECHO result confirmed mitral regurgitations, but an ECG/SPECT study demonstrated no other pathology.

He was referred to Immunology on September 3, 2021 (**Table 1**). During this evaluation, he commented on several occasions that his life had changed completely. During the exam, there was evidence of some word finding difficulty and memory loss but no other abnormalities. The laboratory work up was pertinent for the following (**Table 2**): low IgM, low CD8, and HLA-DR on T cells.

He was started on 50 g IVIG on October 6, 2021 every 2 weeks—which was 254 days after acute COVID—and has had no adverse effects. He reported on March 16, 2022 that the dyspnea was much better and that he no longer needed to take 2- to 3-h naps in the afternoon because of fatigue. He still complained of intermittent left chest pain that was not related to exertion. At approximately 6 months, he reported that he was walking regularly, going to the gym, no longer napping each day, and that the chest pain was gone. He concluded that he was back to his “old self”. The laboratory tests continued to confirm the low IgM, but the CD8 T cells had risen to a normal level at 239 cells/ul, and the persistently elevated high-sensitivity troponin had returned to a normal level for the first time since 2021.

#### Patient 5

A 34-year-old female patient had a past medical history of asthma, migraine headaches, and prolonged recovery from a herpes infection but otherwise had no significant history of infections (**Table 1**). In March 2020, she developed documented COVID with fever, shortness of breath, cough, and myalgias but was ill for only 1 week. In the interim, between the above-mentioned period and January 2022, she was vaccinated with Pfizer vaccine two times, had some reaction to the second shot, but overall was healthy, exercising regularly, and working up to 100 hours per week. In January 2022, COVID recurred, and this time it was much more severe: fever, loss of taste and smell, severe headache, loss of sensation in the feet and tingling in her hands, inability to concentrate, light-headedness, and mild periodic vertigo. In the morning upon waking up, she could not feel her feet and had actually fallen on one occasion. These symptoms persisted, and if anything, some were progressively worse. She stated, “it has changed my life,” and she needed Adderall to keep her going.

She was referred to Immunology on March 16, 2022 (**Table 1**). The result of her physical examination was entirely normal, with the exception that the patient was somewhat tense and hyperactive.

The laboratory workup result was normal except for the IgG2 and IgG3 subclass deficiency (**Table 2**). Based on this and the clinical information, she was selected for a trial of 0.5 g/kg immunoglobulin every 2 weeks. She reported after she had received one treatment of 0.5 g/kg immunoglobulin that, within 2 days, she was back to her old self, but the benefit lapsed in 14 days. Insurance did not approve the every-2-weeks regimen. However, it did approve a regimen of 0.5 g/kg every 3 weeks, and she again has noted a major improvement, now back to fulltime work, and “almost back to normal”.

#### Patient 6

A 39-year-old male patient had a history of chronic sinusitis and fatigue, including three surgical sinus procedures that had not corrected the issue, but he reported no other constitutional symptoms (**Table 1**). However, a biopsy revealed numerous non-necrotizing granulomas; the chest CT demonstrated numerous calcified and noncalcified lymph nodes in the mediastinum and non-caseating nodules throughout both lung fields. ANA and SP-3 levels were elevated. Based on this evidence, a diagnosis of granulomatosis with polyangiitis (Wegener’s) was made, and he was started on methotrexate and low-dose prednisone. There was no evidence of kidney disease. It is important to note that his absolute lymphocyte count was 0.70 and 0.78 on two tests before starting this treatment. When the pulmonary nodules were detected, methotrexate was cancelled and infusions of rituximab were started; the last dose was 6 months before the initiation of IVIG. There continued to be no significant pulmonary symptoms.

On November 8, 2021, a PCR test was found positive, and he developed a progressively severe pulmonary disease consistent with COVID infection, including three hospitalizations for pneumonia. He became very hypoxic and required ventilation. He was treated with high-dose steroids and antibiotics, although no bacterial infections were clearly related. His PCR result remained positive.

He was referred to Immunology on January 26, 2022 (**Table 1**). He had just been discharged from the hospital and had been weaned off from steroids. His severe hypoxia had improved, but he was still on O_2_. The laboratory workup was pertinent for the following (**Table 2**): low plasma immunoglobins: IgA, 73 g/dl; IgM 7, g/dl; IgG, 429 g/dl; absolute lymphocyte count, 0.12 cells/ul; lymphocyte phenotyping: CD3, 60 cells/ul; CD4, 36 cells/ul; CD8, 18 cells/ul; CD19, 0.0 cells/ul; CD16/56, 54 cells/ul; HLA-DR on CD4, 36%; and CD8 T cells, 14%. He had been double-vaccinated and made no anti-spike protein IgG. Because there was immunoglobulin deficiency and severe cellular deficiency, high-dose immunoglobulin was initiated on March 28, 2022 at 0.5 g/kg every 2 weeks—which was 125 days after the initial infection. He has tolerated the regimen, his breathing was very much better, he was no longer on O_2_, and he has fully resumed his normal work and daily activities. Although there has been definite clinical and laboratory improvement, there is still evidence of significant cellular deficiency (**Table 2**).

## Discussion

IVIG has been administered to three overlapping groups of patients: group 1—most frequently in the acute phase of COVID illness (17–22). Although the reports have suggested that it has been generally beneficial, this has not always been true (22). In part, this discrepancy may relate to the dose and time of its administration (17–24) but could also relate to the IVIG product itself (25, 26)—for example, there is emerging evidence that the current IVIG preparations may contain anti-COVID-neutralizing antibodies (27–30) as well as have the capacity to moderate the effect of the initiation of inflammatory cytokines arising in the early acute phase. These characteristics of IVIG may be particularly important for the early treatment of COVID for patients with immune deficiencies (31).

A second group is comprised of those developing disorders or mimicking disorders that persist beyond the acute infectious phase for which IVIG has become an important agent in their treatment (32–37)—for example, the benefit of high-dose IVIG for Guillain–Barre syndrome, chronic inflammatory demyelinating polyneuropathy, multifocal motor neuropathy, and myasthenia gravis has been recognized for years (29, 32) as well as the less well-documented benefit in some other neurological disorders including autoimmune encephalitis (32, 35–37). Importantly, there have now been several reports of the benefit of the early administration of IVIG on the course of COVID-related encephalitis (32, 35, 36).

A third group consists of patients with symptoms that were relatively rare but have become frequently occurring during Long COVID (8, 9, 32, 38–41). Considering the greater-than-12-week time of continuing symptoms to define Long COVID, there are fewer reports of the use of IVIG to treat the various symptoms occurring in this period. Fatigue and cognitive dysfunction remain as major disabilities, and there is a reported increase in severe dysautonomia (38–41). As of this time, the FDA has not approved IVIG for the treatment of these multifaceted disorders,

Most commonly, the IVIG regimen has been to administer 2.0 g/kg or greater of immunoglobulin over a period of 5–7 days and later, if necessary, an additional high dose similar to short courses of IVIG (32). Reference (37) is important because it discusses the initial benefit of a short course of IVIG followed by relapse and successful re-treatment. Patients 1 and 5 and another patient, for whom we have not yet been able to secure approval for the long biweekly regimen, all reported an initial improvement that lapsed within 2 to 3 weeks. Furthermore, there are several conditions in which long-term administration may be necessary (31, 32, 38, 41). Of concern are the thrombotic events that have been reported particularly in the elderly and those with significant co-morbidities.

Because some of our patients had been suffering from Long COVID symptoms for months to years and with the above-mentioned concerns in mind, we chose an IVIG regimen of 0.5 g/kg administered every 2 weeks, with a plan to continue for at least 6 months to 1 year depending on the individual case. With this regimen, there has been no thrombosis, and only two patients have experienced a significant adverse event. One patient required a port for administration, but that became infected. It required 6 weeks of antibiotic therapy, after which a new port was placed without any further incident. A second patient had a history of migraine headaches and developed aseptic meningitis after the first infusion. With better control of her migraines and additional fluids before and during the immunoglobulin infusion, this has not recurred.

It is important to note that all of the patients reported herein were referred because they had persisting symptoms that are now considered as characteristics of Long COVID. Although the immunologic perturbations differed within the group, only one of them was known to be immunodeficient prior to the studies directed at evaluating them for possible treatment for Long COVID. The evaluations discovered IgG subclass deficiency in three patients and low IgM in two patients, evidence for immune activation in four patients, high CD8 in two patients, and low CD8 T cells in one patient. One patient had low immunoglobulins and severe decreases in CD4 T, CD8 T, and CD19 B cells. Of these, three patients were discovered upon further inquiry to have a history of recurrent infections, and one of them was being treated for granulomatous-polyangiitis. Three other patients that were referred for Long COVID also exhibited significant abnormalities in their immune systems but were not treated with IVIG because of financial and logistic issues. They had no history of recurrent infections or autoimmunity. Similar perturbations of the immune system have been recently reported in patients with Long COVID (42).

It is not possible to conclude whether or not the COVID infection was the cause of the immune abnormalities in this group of patients. Low absolute lymphocyte counts are frequently seen in the acute phase of COVID (43), and low levels of IgG have been reported in 20% of patients in one study (44), but few large-scale immunologic reviews, to our knowledge, have been reported in Long COVID patients. Considering that three of this group of Long COVID patients presented with low IgG subclasses and two with low IgM, none of whom had prior knowledge of these deficiencies, it is intriguing to note the recent report (45) that these low levels may predict the risk of Long COVID.

How does this high-dose long-term regimen beneficially affect the central nervous system, peripheral neurological, and cardiac COVID-induced injury? As yet, there is no conclusive evidence for why IVIG can have a beneficial effect on the COVID perturbations, but the role of immunoglobulin Fc interactions with Fc receptors on B cells, NK cells, macrophages, monocytes, dendritic cells, neutrophils, and other hematopoietic cells gives it a wide range of possible interactions (46, 47). Thus, Fc interactions provide a possible explanation for the use of high-dose immunoglobin to treat autoimmune diseases. In addition, IVIG may affect the Fc regions of immunoglobulins and their subclasses. Although this could explain the fact that IVIG can inhibit the level of anti-HLA antibodies and permit transplantation in highly sensitized patients (48, 49), it has also been suggested that this beneficial effect is due to the interaction of idiotypic–anti-idiotypic networks (50). The effect of biweekly high-dose IVIG has been to lower the levels of total IgG and subclass IgG1 in patient 1 (**Table 1**), and that has been associated with a marked improvement in his autoimmune encephalitis. Furthermore, the beneficial effect in patients 1–4 was not clear until at least several doses had been administered. This suggests that one of the effects of long-term high-dose immunoglobulin may be to lower and perhaps stop the production of abnormal antibodies.

A limitation of this small study is that the described benefits are primarily subjective and there is little objective data associated with the outcome. However, it does offer potentially important information that may help guide future studies, namely: (1) IVIG was able to improve Long COVID symptoms months to years after they first occurred, (2) the every-other-week infusion of high-dose IVIG for months was associated with only two significant and reversible adverse events, (3) there may be a number of patients with Long COVID who require a prolonged high level of immunoglobulin to achieve improvement, and (4) it adds further evidence of apparently frequent immune perturbations in patients with Long COVID.

This study does not resolve any of the points listed above but does raise questions that highlight the need for controlled investigations testing the dosages, routes, and duration of immunoglobulin therapy in patients suffering with Long COVID symptoms for extended periods. It should investigate the immunological characteristics that are associated with benefit or failure.

It is worth noting that a plan has been recently proposed as a potential treatment for Long COVID (51), and a clinical trial of 0.4 g/kg for 4 days with a 4-week follow-up is underway (52), but so far it has not been published.

## Data availability statement

The data is in privileged computerized medical records. Requests to access the datasets should be directed to John S. Thompson, jsthom1@uky.edu.

## Ethics statement

Ethical review and approval were not required for the study on human participants in accordance with the local legislation and institutional requirements. The patients/participants provided their written informed consent to participate in this study.

## Author contributions

JT is the primary author, but much of the brief case reports and clinical tests were performed or ordered by the other three authors. All authors contributed to the article and approved the submitted version.
